# Risk stratification after paracetamol overdose using mechanistic biomarkers: results from two prospective cohort studies

**DOI:** 10.1016/S2468-1253(17)30266-2

**Published:** 2017-11-14

**Authors:** James W Dear, Joanna I Clarke, Ben Francis, Lowri Allen, Jonathan Wraight, Jasmine Shen, Paul I Dargan, David Wood, Jamie Cooper, Simon H L Thomas, Andrea L Jorgensen, Munir Pirmohamed, B Kevin Park, Daniel J Antoine

**Affiliations:** a Pharmacology, Toxicology and Therapeutics, University/BHF Centre for Cardiovascular Science, University of Edinburgh, Edinburgh, UK; b MRC Centre for Drug Safety Science, Department of Molecular & Clinical Pharmacology, University of Liverpool, Liverpool, UK; c Department of Biostatistics Institute of Translational Medicine, University of Liverpool, Liverpool, UK; d Clinical Toxicology, Guy's and St Thomas' NHS Foundation Trust, London, UK; e Faculty of Life Sciences and Medicine, King's College London, London, UK; f Emergency Department, Aberdeen Royal Infirmary, Aberdeen, UK; g Medical Toxicology Centre, Institute of Cellular Medicine, Newcastle University, Newcastle, UK

## Abstract

**Background:**

Paracetamol overdose is common but patient stratification is suboptimal. We investigated the usefulness of new biomarkers that have either enhanced liver specificity (microRNA-122 [miR-122]) or provide mechanistic insights (keratin-18 [K18], high mobility group box-1 [HMGB1], and glutamate dehydrogenase [GLDH]). The use of these biomarkers could help stratify patients for their risk of liver injury at hospital presentation.

**Methods:**

Using data from two prospective cohort studies, we assessed the potential for biomarkers to stratify patients who overdose with paracetamol. We completed two independent prospective studies: a derivation study (MAPP) in eight UK hospitals and a validation study (BIOPAR) in ten UK hospitals. Patients in both cohorts were adults (≥18 years in England, ≥16 years in Scotland), were diagnosed with paracetamol overdose, and gave written informed consent. Patients who needed intravenous acetylcysteine treatment for paracetamol overdose had circulating biomarkers measured at hospital presentation. The primary endpoint was acute liver injury indicating need for continued acetylcysteine treatment beyond the standard course (alanine aminotransferase [ALT] activity >100 U/L). Receiver operating characteristic (ROC) curves, category-free net reclassification index (cfNRI), and integrated discrimination index (IDI) were applied to assess endpoint prediction.

**Findings:**

Between June 2, 2010, and May 29, 2014, 1187 patients who required acetylcysteine treatment for paracetamol overdose were recruited (985 in the MAPP cohort; 202 in the BIOPAR cohort). In the derivation and validation cohorts, acute liver injury was predicted at hospital presentation by miR-122 (derivation cohort ROC–area under the curve [AUC] 0·97 [95% CI 0·95–0·98]), HMGB1 (0·95 [0·93–0·98]), and full-length K18 (0·95 [0·92–0·97]). Results were similar in the validation cohort (miR-122 AUC 0·97 [95% CI 0·95–0·99], HMGB1 0·98 [0·96–0·99], and full-length K18 0·93 [0·86–0·99]). A combined model of miR-122, HMGB1, and K18 predicted acute liver injury better than ALT alone (cfNRI 1·95 [95% CI 1·87–2·03], p<0·0001 in the MAPP cohort; 1·54 [1·08–2·00], p<0·0001 in the BIOPAR cohort).

**Interpretation:**

Personalised treatment pathways could be developed by use of miR-122, HMGB1, and full-length K18 at hospital presentation for patient stratification. This prospective study supports their use for hepatic safety assessment of new medicines.

**Funding:**

Edinburgh and Lothians Health Foundation, UK Medical Research Council.

## Introduction

Paracetamol is a safe analgesic drug when taken at therapeutic doses. However, in overdose, paracetamol is hepatotoxic and is the most common cause of acute liver failure in the USA and Europe.^1^, ^2^ After overdose, the metabolite N-acetyl-p-benzoquinone imine (NAPQI) is generated in excess, which depletes glutathione (GSH) and leads to oxidative stress and hepatocyte death, predominately by necrosis.^3^ Cell death releases intracellular molecules into the blood to produce changes in circulating protein and RNA.^4^

The current antidote, acetylcysteine, prevents liver injury by replenishing GSH if administered within a few hours of overdose.^5^ The decision to commence treatment with acetylcysteine after a single overdose is based on the reported dose ingested and a timed blood paracetamol concentration, which is interpreted using a binary treat or no treat nomogram with the treatment threshold at a level of low risk. Even with this conservative approach there are patients who develop acute liver injury. Current markers, serum alanine aminotransferase (ALT) activity and paracetamol concentration, lack sensitivity and specificity when measured soon after overdose such as at initial presentation to hospital.^6^, ^7^, ^8^ These limitations are further compounded in staggered overdose, for which there is an increased acute liver injury risk but for which treatment nomograms are not recommended.^9^, ^10^ Acute liver injury results in prolonged hospital admission for continued acetylcysteine therapy (with concomitant prolonged occupancy of acute hospital beds) and, in severe cases, might result in acute liver failure and even the need for liver transplantation to avoid death (fortunately these life-threatening clinical scenarios are rare with prompt acetylcysteine treatment). Targeted therapies that reduce cell death and inflammation^11^ and aid tissue regeneration^12^ are in development, but they are still some way from routine clinical implementation.

**Research in context**

**Evidence before this study**

Paracetamol overdose is a common reason for hospital admission. The decision to treat with the antidote acetylcysteine is based on the reported dose of drug ingested, a timed blood paracetamol concentration, and whether there is liver injury, which is reported predominately by serum alanine aminotransferase (ALT) activity. We searched PubMed with no restrictions using the keywords “biomarker” AND “paracetamol” OR “acetaminophen” AND “liver” AND “human” AND “hospital admission”, which yielded 101 papers by the end of the search on Dec 16, 2016. Review of these papers highlighted an unmet need for new treatment pathways that are informed by an enhanced ability to identify patients who will develop liver injury despite current treatment. The results of this search included our 2013 proof-of-concept study, which showed that a panel of new biomarkers stratified patients with paracetamol overdose by their risk of liver injury at hospital presentation. This study included 129 patients who had an acute overdose and highlights the need for multicentre prospective studies that include all patterns of overdose.

**Added value of this study**

We, and others, have identified sensitive and specific biomarkers of paracetamol hepatotoxicity that have either enhanced liver specificity (microRNA-122 [miR-122]) or provide mechanistic insights (keratin-18 [K18], high mobility group box-1 [HMGB1], and glutamate dehydrogenase [GLDH]). HMGB1 is a mediator of toxicity and potential drug target. This study tested the usefulness of these biomarkers prospectively in derivation and validation overdose cohorts. We showed that these new biomarkers accurately identify patients who will get liver injury despite current guideline-based clinical treatment. The performance of the markers is maintained across the different paracetamol overdose clinical phenotypes.

**Implications of all the available evidence**

With regard to paracetamol overdose, future clinical trials should incorporate measurement of miR-122, HMGB1, and K18 because these markers identify patients, at first attendance to hospital, who require additional treatment to prevent liver injury. Beyond paracetamol overdose, this study confirms that these markers are more sensitive than current liver injury markers. They should be added into the assessment of hepatic safety for new medicines in early phase clinical trials.

Stratified pathways that selectively target treatments to patients who stand to benefit would be ideal. However, there is an unmet need for accurate biomarkers that predict liver injury that will not be prevented by standard acetylcysteine treatment soon after overdose.^6^, ^7^, ^8^ These biomarkers should also be useful in the context of a staggered overdose, a scenario with increased acute liver injury risk but for which treatment nomograms are not applicable.^9^, ^10^

Preclinical studies of paracetamol-induced acute liver injury have identified the liver-enriched microRNA-122 (miR-122), high mobility group box-1 (HMGB1), keratin-18 (K18; both caspase-cleaved and full-length), and glutamate dehydrogenase (GLDH) as sensitive predictors of subsequent hepatotoxicity.^13^, ^14^ In 129 patients who had ingested a single paracetamol overdose (no staggered overdoses were included) we showed in a proof-of-concept study^15^ that miR-122, HMGB1, and full-length K18 can identify acute liver injury on hospital admission at a time when currently used markers of liver injury (ie, ALT concentration) were still normal. miR-122 provides enhanced hepatic specificity over all the current biomarkers.^16^ HMGB1 is reflective of cell necrosis and activated immune cells.^11^ Inhibition of HMGB1 in rodent models attenuates paracetamol toxic effects, indicating that it is a mediator of injury.^11^ Cell apoptosis is reported by caspase-cleaved K18, whereas full-length K18 reports necrosis.^13^ GLDH is a marker of mitochondrial dysfunction.^17^ These markers also have the potential to aid the process of hepatic safety assessment in preclinical and clinical drug development. This has been recognised with regulatory support for further qualification of miR-122, HMGB1, K18, and GLDH being given by the US Food and Drug Administration (FDA) and the European Medicines Agency (EMA) across the spectrum of drug-induced liver injury.^18^, ^19^

The objective of this study was to explore the ability of these new mechanistic biomarkers to stratify patients by risk of subsequent liver injury in two prospectively recruited cohorts of patients with paracetamol overdose who faithfully represented the spectrum of clinical presentations.

## Methods

### Study design and participants

We report the outcomes of two prospective cohort studies: Markers and Paracetamol Poisoning (MAPP) and Biomarkers of Paracetamol Hepatotoxicity (BIOPAR). Both were done in compliance with the Declaration of Helsinki and Good Clinical Practice guidelines and reported according to the Standards for Reporting Diagnostic Accuracy.^20^, ^21^

For the MAPP study, adults (≥16 years in Scotland, ≥18 years in England) were recruited when research staff were available and if the patient had capacity to provide informed consent as per the study inclusion criteria from eight hospitals in the UK. Research nurses identified participants on admission to hospital. The inclusion criteria were: a history of paracetamol overdose that the treating clinician judged to warrant treatment with intravenous acetylcysteine as per UK guidelines, a first blood sample obtained within 24 h of paracetamol ingestion, and for patients to have the capacity to consent. All patterns of paracetamol overdose were eligible for inclusion (ie, early presenting acute overdose or late presenting acute and staggered oversode). Staggered overdose was defined as ingestion over 2 h or more. The exclusion criteria were detention under the Mental Health Act; documented cognitive impairment; inability to provide informed consent for any reason; or an unreliable overdose history. Full written informed consent was obtained from every participant, and ethical approval was from the South East Scotland Research Ethics Committee and the East of Scotland Research Ethics Committee via the South East Scotland Human Bioresource.

For the BIOPAR study, adults (≥18 years) were recruited by convenience sampling from ten UK hospitals. The inclusion criteria were willingness to provide informed consent and diagnosis with paracetamol overdose (>4 g in 24 h). All patterns of paracetamol overdose were eligible for inclusion. Staggered ingestion was defined as over 2 h or more. Patients were excluded if they were unable to consent or not suitable for participation, as determined by the local investigator. Full written informed consent was obtained from every participant and ethical approval was from the North West Centre of Research Ethics Committee.

### Procedures

In MAPP and BIOPAR, demographic information was recorded for study participants and the blood sample taken at first presentation to hospital was stored at −80°C as plasma or serum. All blood results from the first hospital admission were recorded (paracetamol, alkaline phosphatase, γ-glutamyl transferase, bilirubin, creatinine, and ALT concentration, prothrombin time, and international normalised ratio [INR]). Data were also obtained regarding the pattern of overdose, time of overdose, and other drugs ingested.

The reference standard of injury was ALT concentration. miR-122, HMGB1, caspase-cleaved K18, full-length K18, and GLDH were measured in the admission blood sample, as previously described,^15^ with miR-122 measured by PCR and other markers measured by ELISA; each biomarker was measured in each sample in duplicate. miR-122 concentration was expressed with reference to the circulating microRNA let-7d as the internal microRNA normaliser. Investigators measuring the novel biomarkers were masked to the details of patient history and other biochemistry results. Assay development and validation have been described previously.^13^, ^22^

### Outcomes

The primary endpoint was acute liver injury, predefined as ALT greater than 100 U/L (the UK criteria for the use of additional acetylcysteine beyond the standard 21 h course). Secondary endpoints were peak ALT of more than 1000 U/L and liver synthetic dysfunction (INR>1·5).

### Statistical analysis

The recruitment target was 1000 patients for the derivation (MAPP) cohort, with 8% of patients anticipated to exhibit the primary endpoint.^23^ On this basis, the denominator would be 920 for calculating the biomarker negative predictive value and 80 for the positive predictive value. Given that the aim of this study was to identify the success of these biomarkers and tests for the purposes of identifying the primary endpoint, the denominator for determining the negative and positive predictive values was unknown. However, a broad range of denominators was considered based on what might be clinically plausible considering these assumptions. This included between 750 and 950 patients for the negative predictive value and between 50 and 250 patients for the positive predictive value. Across a broad range of possible negative predictive values (50–90%), the 95% CI range for the negative predictive value would be ±1·9% to ±3·6%, indicating very precise estimation. The 95% CI range for the positive predictive value would range from ±3·7% to ±13·9%, indicating acceptable precision.

Initially, receiver operating characteristic (ROC) curves were derived from values at first presentation. The areas under ROC curves (AUCs) were compared between biomarkers by the methods developed previously using Graphpad Prism version 7.^15^ We used R, version 2.1.2.1, for multivariable logistic regression analyses and to assess the discriminative ability of a combination of biomarkers for predicting the primary endpoint. Singular biomarkers that were significant in univariable logistic regression were entered into forward, backward, and stepwise selection processes. The results of these selection processes were compared to discern the maximal model.

We investigated the ability of our biomarkers to predict the later development of acute liver injury with category-free net reclassification index (cfNRI) and integrated discrimination index (IDI) analyses.^24^ Because there are no established risk thresholds for acute liver injury, cfNRI was used as opposed to the standard net reclassification index (NRI). To discern improved performance, cfNRI or IDI requires a current standard model to compare with a proposed maximal model. For acute liver injury, the current standard model is the use of ALT on presentation to hospital and this was therefore compared with the maximal model identified by the variable selection processes. To apply cfNRI or IDI, we used the Harrell miscellaneous (Hmisc) R package, version 4.0-2.

### Role of the funding source

The funders of the study had no role in study design, data collection, data analysis, data interpretation, or writing of the report. The corresponding author had full access to all the data in the study and had final responsibility for the decision to submit for publication.

## Results

Between June 2, 2010, and May 29, 2014, 1187 patients who required acetylcysteine treatment for paracetamol overdose were recruited (985 in the MAPP cohort; 202 in the BIOPAR cohort; figure, appendix p 3). Demographic data and clinical chemistry parameters are presented in table 1. No patients died or required liver transplantation in either cohort. Alcohol was co-ingested in 473 (48%) of 985 patients in the MAPP cohort and in 105 (52%) of 202 patients in the BIOPAR cohort. There were no missing data in either cohort.

**Figure:**
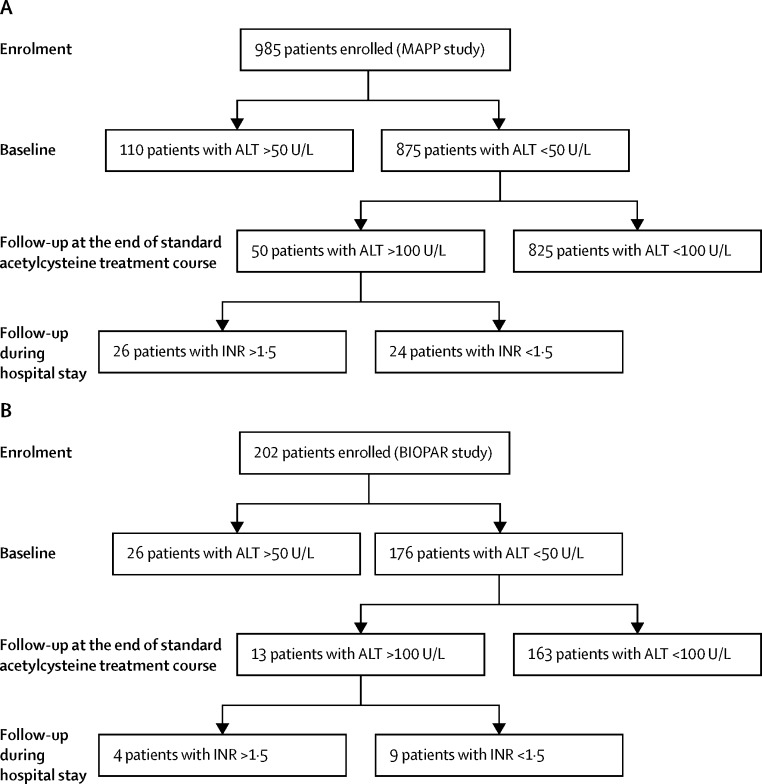
Study profile Summary of the patient outcome pathways for all patients prospectively recruited to the (A) derivation (MAPP) and (B) validation (BIOPAR) cohorts. ALT=alanine aminotransferase. INR=international normalised ratio.

**Table 1:** Baseline characteristics by overdose type

		**Derivation cohort (MAPP)**			**Validation cohort (BIOPAR)**		
		Acute (n=672)	Staggered (n=237)	Unknown (n=76)	Acute (n=137)	Staggered (n=64)	Unknown (n=1)
Sex							
	Male	232 (35%)	106 (45%)	28 (37%)	66 (48%)	28 (44%)	0
	Female	440 (65%)	131 (55%)	48 (63%)	71 (52%)	36 (56%)	1 (100%)
Age, years		33 (20–45)	37 (25–46)	33 (22–43)	29 (23–46)	29 (22–48)	56
Body-mass index, kg/m^2^		26·4 (22·1–29·0)	24·9 (21·0–27·9)	25·8 (21·9–29·1)	25·3 (20·4–28·0)	25·8 (22·0–28·3)	25·7
Amount of paracetamol ingested, g		15 (8–20)	15 (9–25)	13 (9–20)	22 (16–40)	28 (16–48)	16
Time from ingestion to first blood sample, h		4 (4–7)	6 (3–13)	Unknown	6 (4–10)	8 (4–14)	Unknown
Admission paracetamol concentration, mg/L		100 (52–154)	27 (10–73)	73 (45–139)	114 (75–153)	38 (5–104)	1
Admission ALP, U/L		72 (59–88)	74 (62–92)	68 (53–79)	80 (63–92)	75 (58–83)	71
Admission serum creatinine, μmol/L		65 (58–73)	67 (66–76)	69 (62–77)	72 (61–85)	80 (70–91)	68
ALT							
	Admission ALT, U/L	18 (14–28)	20 (15–35)	18 (12–23)	21 (16–23)	25 (17–36)	43
	Number with admission ALT <ULN	598	207	70	121	54	1
	Number with admission ALT >100 U/L	38	11	4	8	5	0
	Number with admission ALT >1000 U/L	6	2	2	3	3	0
	Number with peak ALT >100 U/L	71	24	10	17	9	0
	Number with peak ALT >1000 U/L	17	7	4	7	4	0
INR							
	Admission INR	1 (1·0–1·1)	1 (0·9–1)	1 (1–1·1)	1 (0·9–1)	1 (1·0–1·1)	0·9
	Number with admission INR <1·5	658	229	75	133	60	1
	Number with admission INR >1·5	14	8	1	4	4	0

Data are median (IQR) or n (%). ALT=alanine aminotransferase. ALP=alkaline phosphatase. GGT=gamma glutamyl transpeptidase. INR=international normalised ratio. ULN=upper limit of normal (cutoff 50 U/L).

There were significant correlations between peak hospital stay, activity of ALT, and concentrations of all biomarkers on first presentation to hospital in both the derivation and validation cohorts (appendix pp 1–2). These analyses were also supported by ROC analysis showing the associations between biomarker concentrations at presentation to hospital and peak ALT of more than 100 U/L (table 2). In the derivation cohort, ROC–AUC values to predict ALT greater than 100 U/L were: 0·97 (95% CI 0·95–0·98) for miR-122, 0·95 (0·93–0·98) for HMGB1, and 0·95 (0·92–0·97) for full-length K18. These values were similar for the validation cohort (miR-122 0·97 [0·95–0·99], HMGB1 0·98 [0·96–0·99], 0·93 [0·86–0·99]). The ROC analysis also showed association between biomarker concentrations and peak ALT of more than 1000 U/L (appendix pp 5–6).

**Table 2:** Novel biomarkers accurately predicted peak ALT of more than 100 U/L after paracetamol overdose

	**Derivation cohort (MAPP) n/N=105/985**						**Validation cohort (BIOPAR) n/N=26/202**					
	ROC–AUC	p value	Specificity	Sensitivity	PPV	NPV	ROC–AUC	p value	Specificity	Sensitivity	PPV	NPV
ALT	0·84 (0·79–0·89)	<0·0001	0·95	0·52 (0·42–0·62)	53·9	94·3	0·81 (0·71–0·92)	<0·0001	0·95	0·50 (0·29–0·70)	61·9	92·8
Paracetamol concentration	0·56 (0·49–0·62)	0·0569	0·95	0·09 (0·04–0·16)	10·4	88·6	0·50 (0·36–0·63)	0·9928	0·95	0·15 (0·04–0·35)	33·3	88·4
miR-122	0·97 (0·95–0·98)	<0·0001	0·95	0·79 (0·70–0·87)	65·4	97·4	0·97 (0·95–0·99)	<0·0001	0·95	0·84 (0·65–0·95)	71·0	97·6
HMGB1	0·95 (0·93–0·98)	<0·0001	0·95	0·82 (0·73–0·88)	65·7	97·8	0·98 (0·96–0·99)	<0·0001	0·95	0·81 (0·61–0·93)	70·0	97·1
Full-length K18	0·95 (0·92–0·97)	<0·0001	0·95	0·56 (0·46–0·66)	57·3	94·8	0·93 (0·86–0·99)	<0·0001	0·95	0·54 (0·33–0·73)	70·0	97·1
Caspase-cleaved K18	0·84 (0·78–0·89)	<0·0001	0·95	0·65 (0·56–0·75)	61·1	95·9	0·87 (0·78–0·97)	<0·0001	0·95	0·69 (0·48–0·86)	66·7	95·4
GLDH	0·86 (0·82–0·90)	<0·0001	0·95	0·58 (0·48–0·68)	58·1	95·0	0·83 (0·74–0·93)	<0·0001	0·95	0·54 (0·33–0·73)	63·6	93·3

ROC–AUC (95% CI), sensitivity at 95% specificity (95% CI), and PPV and NPV were calculated to identify the potential of novel and established stratification biomarkers to predict the development of acute liver injury (ALT ≥100 U/L). ROC=receiver operator characteristic. AUC=area under the curve. PPV=positive predictive value. NPV=negative predictive value. ALT=alanine aminotransferase. HMGB1=high mobility group box-1. GLDH=glutamate dehydrogenase.

To establish whether the biomarkers had enhanced sensitivity we focused on the 875 patients from the derivation cohort with a normal ALT activity (<50 U/L) and INR (<1·5) at first presentation to hospital (figure). miR-122, HMGB1, and both K18 molecular forms were measured in all patients and were significantly higher in patients who developed subsequent acute liver injury (appendix p 3). These biomarkers performed with a high degree of sensitivity and specificity in identifying subsequent acute liver injury (ie, an increase in ALT >100 U/L) in both the derivation and validation cohorts (table 3), or an ALT of more than 1000 U/L (appendix p 6). For patients with acute overdose, miR-122, HMGB1, and K18 maintained sensitivity and specificity when patients were censored by time from overdose to initiation of acetylcysteine (less than 8 h or 8 h and greater) in both the derivation and validation cohorts (appendix p 7).

**Table 3:** Accuracy of biomarkers to predict peak ALT of more than 100 U/L in patients with normal ALT and INR at hospital presentation

	**Derivation cohort (MAPP) n/N=40/875**						**Validation cohort (BIOPAR) n/N=13/176**					
	ROC–AUC	p value	Specificity	Sensitivity	PPV	NPV	ROC–AUC	p value	Specificity	Sensitivity	PPV	NPV
ALT	0·61 (0·51–0·68)	0·02015	0·95	0·22 (0·11–0·36)	22·0	95·3	0·63 (0·50–0·76)	0·0071	0·95	0·29 (0·15–0·41)	8·8	92·9
Paracetamol concentration	0·58 (0·51–0·66)	0·03762	0·95	0·10 (0·03–0·22)	6·9	94·4	0·59 (0·40–0·69)	0·0079	0·95	0·31 (0·09–0·51)	33·3	94·5
miR-122	0·96 (0·93–0·99)	<0·0001	0·95	0·84 (0·71–0·93)	48·8	98·9	0·97 (0·94–1·00)	<0·0001	0·95	0·92 (0·64–0·99)	70·5	99·4
HMGB1	0·94 (0·89–0·98)	<0·0001	0·95	0·88 (0·76–0·95)	45·8	99·2	0·98 (0·97–1·00)	<0·0001	0·95	0·92 (0·64–0·99)	54·5	99·4
Full-length K18	0·94 (0·89–0·99)	<0·0001	0·95	0·88 (0·76–0·95)	54·3	99·2	0·93 (0·81–1·00)	<0·0001	0·95	0·85 (0·55–0·98)	61·1	98·7
Caspase-cleaved K18	0·79 (0·70–0·88)	<0·0001	0·95	0·60 (0·45–0·74)	51·8	97·3	0·80 (0·63–0·97)	0·0003	0·95	0·69 (0·39–0·90)	52·9	97·5
GLDH	0·74 (0·67–0·82)	<0·0001	0·95	0·34 (0·21–0·49)	27·3	95·7	0·65 (0·49–0·81)	0·0068	0·95	0·15 (0·02–0·45)	20·1	93·3

ROC–AUC (95% CI), sensitivity at 95% specificity (95% CI), PPV, and NPV were calculated to assess the potential of novel and established stratification biomarkers to predict the development of acute liver injury. ROC=receiver operator characteristic. AUC=area under the curve. PPV=positive predictive value. NPV=negative predictive value. ALT=alanine aminotransferase. HMGB1=high mobility group box-1. GLDH=glutamate dehydrogenase.

All biomarkers and patient characteristics were included in the multivariable logistic regression analyses to assess improvement in prognostic ability. Forward, backward, and stepwise selection processes all confirmed that the maximal prognostic model consisted of miR-122, HMGB1, full-length K18, and caspase-cleaved K18 (cfNRI 1·95 [95% CI 1·87–2·03], p<0·0001 in the MAPP cohort and 1·54 [1·08–2·00], p<0·0001 in the BIOPAR cohort; appendix p 8). The added prognostic ability of the maximal prognostic model to an ALT-only model was supported by the results of cfNRI and IDI (appendix p 8). To assess whether the maximal prognostic model components could be reduced by assessment of the subsequent impact on cfNRI and IDI, the biomarkers within the model derived from the derivation cohort were ordered by the magnitude of their standardised coefficient thus: miR-122 (standardised β coefficient 149·7), full-length K18 (8·7), caspase-cleaved K18 (8·0), then HMGB1 (4·3). With miR-122 alone, 45 (90%) of 50 patients in the derivation cohort with acute liver injury were correctly identified at first presentation. In patients without acute liver injury, 735 (89%) of 825 patients in the derivation cohort were correctly identified (appendix p 9). With the addition of full-length K18, caspase-cleaved K18, and HMGB1, 49 (98%) of 50 patients with acute liver injury were correctly identified at first presentation. In patients without acute liver injury, 824 (99·9%) of 825 patients were correctly identified with this combination of biomarkers (appendix p 9). When the maximal prognostic model derived from the derivation cohort was assessed in the validation cohort using cfNRI and IDI, it continued to provide robust stratification of patients by risk of subsequent acute liver injury. Similar to the derivation cohort, miR-122 alone identified nine (69%) of 13 patients in the validation cohort with subsequent acute liver injury at first presentation to hospital. In patients without subsequent acute liver injury, all of the 163 patients in the validation cohort were correctly identified by miR-122 alone (appendix p 9). With the addition of full-length K18, caspase-cleaved K18, and HMGB1, ten (77%) of 13 patients with subsequent acute liver injury were correctly identified at first presentation (appendix p 9).

HMGB1 had the highest ROC–AUC, most favourable positive and negative predictive values, and the highest sensitivity at 95% specificity for the prediction of the secondary endpoint hepatic synthetic dysfunction (INR >1·5) in both cohorts (table 4). Multivariable logistic regression analyses confirmed the maximal prognostic model for elevation in INR consisted only of HMGB1 (appendix p 4). For the analysis of HMGB1 in all patients in the derivation cohort who presented to hospital with normal liver function tests, 21 (66%) of 32 cases were correctly identified as having a subsequent increase in INR. In patients who did not develop an increase in INR, 816 (97%) of 843 patients in the derivation cohort were correctly identified. In the validation cohort, three (50%) of six patients were correctly identified as having a subsequent increase in INR despite normal liver function tests at first presentation to hospital. In patients who did not develop an increase in INR, 163 (96%) of 170 were correctly identified.

**Table 4:** Biomarkers accurately predict peak INR of more than 1·5 in patients who had a normal ALT and INR at hospital presentation

	**Derivation cohort (MAPP) n/N=26/875**						**Validation cohort (BIOPAR) n/N=4/176**					
	ROC–AUC	p value	Specificity	Sensitivity	PPV	NPV	ROC–AUC	p value	Specificity	Sensitivity	PPV	NPV
ALT	0·55 (0·39–0·72)	0·473	0·95	0·23 (0·09–0·44)	70·0	52·5	0·57 (0·31–0·80)	0·369	0·95	0·25 (0·12–0·45)	0·0	63·6
Paracetamol concentration	0·53 (0·36–0·70)	0·699	0·95	0·00 (0·00–0·15)	0·0	45·8	0·55 (0·40–0·79)	0·773	0·95	0·00 (0·00–0·13)	0·0	66·7
miR-122	0·73 (0·59–0·88)	0·0043	0·95	0·46 (0·27–0·66)	92·3	62·2	0·75 (0·41–1·00)	0·016	0·95	0·50 (0·31–0·67)	66·7	80·0
HMGB1	0·94 (0·88–1·00)	<0·0001	0·95	0·88 (0·70–0·98)	92·0	88·0	0·90 (0·73–1·00)	0·025	0·95	0·65 (0·50–0·88)	75·0	88·9
Full-length K18	0·81 (0·69–0·93)	0·0001	0·95	0·27 (0·11–0·48)	87·5	54·8	0·86 (0·65–1·00)	0·045	0·95	0·25 (0·10–0·43)	50·0	72·3
Caspase-cleaved K18	0·82 (0·70–0·94)	<0·0001	0·95	0·27 (0·11–0·48)	77·8	53·7	0·81 (0·55–1·00)	0·008	0·95	0·25 (0·10–0·43)	50·0	72·3
GLDH	0·66 (0·51–0·82)	0·042	0·95	0·42 (0·23–0·63)	73·3	57·1	0·58 (0·26–0·88)	0·643	0·95	0·25 (0·10–0·44)	0·0	66·7

ROC–AUC (95% CI), sensitivity at 95% specificity (95% CI), PPV, and NPV were calculated to identify the potential of novel and established stratification biomarkers to predict the development of hepatic dysfunction. INR=international normalised ratio. ROC=receiver operator characteristic. AUC=area under the curve. PPV=positive predictive value. NPV=negative predictive value. ALT=alanine aminotransferase. HMGB1=high mobility group box-1. GLDH=glutamate dehydrogenase.

After staggered overdose, miR-122, HMGB1, and K18 were significantly higher in patients who developed acute liver injury than those who did not in both the MAPP (miR-122 0·16 *vs* 60·2, HMGB1 1·01 ng/mL *vs* 6·98 ng/mL, full-length K18 289·6 U/L *vs* 851·9 U/L, all p<0·0001) and BIOPAR cohorts (miR-122 0·14 *vs* 28·1, p<0·0001; HMGB1 1·07 ng/mL *vs* 4·9 ng/mL, p<0·0001; full-length K18 279·5 U/L *vs* 698·7 U/L, p=0·082; table 5). Again, in both the derivation and validation cohorts, ROC analysis showed high sensitivity and specificity with regard to identifying acute liver injury at first presentation to hospital, with miR-122 and HMGB1 having ROC–AUC values of 1 (table 5). Finally, in the derivation cohort there were 76 patients with an unknown pattern of overdose. In these patients, ROC–AUC values for miR-122 (0·93, 95% CI 0·85–1·00), HMGB1 (0·89, 0·75–1·00), and K18 (0·85, 0·59–1·00) showed a high sensitivity and specificity for acute liver injury. Only one patient had an unknown pattern of oversode in the validation cohort, so it was not possible to verify the findings in this cohort.

**Table 5:** Novel biomarkers accurately predict peak ALT of more than 100 U/L in patients who presented with a staggered paracetamol overdose with normal ALT and INR at hospital presentation

	**Derivation cohort (MAPP) n/N=13/207**						**Validation cohort (BIOPAR) n/N=4/54**					
	ROC–AUC	p value	Specificity	Sensitivity	PPV	NPV	ROC–AUC	p value	Specificity	Sensitivity	PPV	NPV
ALT	0·63 (0·48–0·77)	0·1342	0·95	0·38 (0·14–0·68)	29·4	95·8	0·57 (0·23–0·91)	0·6203	0·95	0·50 (0·22–0·71)	50·0	96·0
Paracetamol concentration	0·57 (0·38–0·77)	0·3451	0·95	0·15 (0·02–0·45)	20·0	94·5	0·67 (0·52–0·81)	0·1019	0·95	0·25 (0·13–0·55)	25·0	94·0
miR-122	1·00 (1·00–1·00)	<0·0001	0·95	1·00 (0·75–1·00)	54·2	100·0	1·00 (1·00–1·00)	<0·0001	0·95	1·00 (0·75–1·00)	66·7	100·0
HMGB1	1·00 (1·00–1·00)	<0·0001	0·95	1·00 (0·75–1·00)	59·1	100·0	0·98 (0·94–1·00)	<0·0001	0·95	1·00 (0·75–1·00)	57·2	100·0
Full-length K18	0·99 (0·98–1·00)	<0·0001	0·95	0·92 (0·64–0·99)	54·5	99·4	0·76 (0·37–1·00)	0·0800	0·95	0·75 (0·44–0·92)	50·0	97·9
Caspase-cleaved K18	0·77 (0·59–0·95)	0·0011	0·95	0·62 (0·32–0·86)	44·4	97·3	0 63 (0·22–1·00)	0·3905	0·95	0·50 (0·22–0·74)	40·0	95·9
GLDH	0·78 (0·62–0·93)	0·0009	0·95	0·53 (0·25–0·81)	36·8	96·8	0·70 (0·47–0·93)	0·1757	0·95	0·25 (0·10–0·46)	25·0	94·0

ROC–AUC (95% CI), sensitivity at 95% specificity (95% CI), PPV, and NPV predictive values were calculated to identify the potential of novel and established stratification biomarkers to predict the development of acute liver injury. INR=international normalised ratio. ROC=receiver operator characteristic. AUC=area under the curve. PPV=positive predictive value. NPV=negative predictive value. ALT=alanine aminotransferase. HMGB1=high mobility group box-1. GLDH=glutamate dehydrogenase.

## Discussion

In these prospective cohort studies we have shown that a panel of novel biomarkers was able to identify patients at risk of subsequent liver injury at first presentation to hospital. miR-122, HMGB1, and K18 had superior sensitivity compared with the current gold standard marker (ALT). Furthermore, we have shown that HMGB1 predicted liver synthetic dysfunction. We propose that this combination of biomarkers could be used for stratification of patients for their risk of liver injury and therefore targeting of preventive novel treatment pathways.

The derivation cohort recruited 985 patients across eight hospitals, including a range of paracetamol overdose presentations (early presenting, late presenting, and staggered ingestions) that faithfully reflect routine clinical practice. Importantly, acute alcohol consumption, which is common with paracetamol overdose, does not significantly affect the circulating concentration of miR-122, HMGB1, or K18 and therefore is not a potential confounding factor.^25^

The best predictive model was composed of miR-122, HMGB1, and the K18 isoforms. Addition of GLDH, a putative marker of mitochondrial toxicity, all current biomarkers (ALT, INR, plasma paracetamol concentration), and all recorded patient clinical characteristics (eg, dose ingested and time to treatment) did not improve the specificity or sensitivity of the model. When ranked by their standardised contribution to the model, miR-122 was the best predictor of ALT increase, probably because of the enhanced liver specificity of miR-122 compared with ALT, its bioanalytical sensitivity of detection, and because microRNAs can be actively secreted from dying or stressed hepatocytes with their membrane integrity intact.^26^ The improved tissue specificity of miR-122 compared with ALT is supported by the observation that increases in ALT associated with muscle injury are not accompanied by concomitant increases in miR-122.^27^

Despite our findings, the following limitation warrants consideration. This study was an observational cohort study with the overall event rate of abnormal ALT (ie, >100 U/L) being 10·7% in the derivation cohort and 12·9% in the validation cohort. This low event rate could bias model performance. However, the event rate with regard to our primary endpoint is consistent with other studies^28^ and is similar to data we obtained from auditing all consecutive patients admitted to hospital with paracetamol overdose in Edinburgh over 3 years (ALT >100 U/L in 164 [10%] of 1641 patients; J W Dear, University of Edinburgh, unpublished data). Furthermore, the number of patients recruited was guided by a power calculation that incorporated a lower event rate than that actually observed (8%). Therefore, the data presented in this study are relevant to the common clinical challenge of treating an unselected population of patients with paracetamol overdose. This large population of people who present to the hospital and require intervention (about 50 000 per year in the UK) predominantly use health service resources by needing treatment with acetylcysteine in hospital emergency beds rather than by needing escalation to critical care. Future studies are needed to define the ability of these markers to predict rarer outcomes such as liver failure and death.

Our primary endpoint was an increase in ALT to more than 100 U/L and is lower than published case definitions for drug-induced liver injury.^29^ However, this cutoff is used clinically to indicate the need for further acetylcysteine treatment in the UK with concomitant prolongation of hospital stay. Additionally, this ALT cutoff is evidence based. The increases in ALT concentrations that accompany paracetamol overdose have been previously described.^30^ Green and colleagues^30^ showed that 91 (97%) of 94 patients with a peak ALT concentration of more than 1000 U/L (indicative of hepatotoxicity) also had an ALT concentration of more than 100 U/L at the end of the standard 21 h acetylcysteine treatment. It is therefore unlikely that substantial liver injury will occur if ALT is less than 100 U/L, and this finding supports the basis of the UK clinical guidance. Although other countries have different criteria for identifying whether or not it is safe to discontinue acetylcysteine treatment, multiples of the upper limit of normal (50 U/L) of ALT are still used. For example, acetylcysteine is continued if ALT is greater than 50 U/L in Australia on the basis of literature evidence^30^ that substantial liver injury is unlikely if ALT concentrations are less than 100 U/L; however, 50 U/L or greater is still above the limit of normal, so this conservative approach is still used. However, we acknowledge the ALT increase to 100 U/L used in our study was modest and did not result in life-threatening disease and requires investigation in other cohorts worldwide. Additionally, our findings show that biomarker performance was also maintained in both the derivation and validation cohorts when a more substantial increase in ALT activity of more than 1000 U/L was assessed. It is also important to note that even substantive increases in ALT activity are not useful for prognosis in patients with established acute liver injury and so ALT is not part of prognostic models such as the King's College Criteria (KCC)^31^ and MELD.^32^ By contrast, INR is established as a biomarker of patient prognosis and is a core component of KCC and MELD.

In our study we also analysed INR increases as a secondary endpoint and showed that only HMGB1 at first presentation to hospital predicted a subsequent INR of more than 1·5. This was shown by ROC analysis and multivariable logistic regression analysis in the derivation cohort and replicated in the validation cohort. Lower patient numbers reached this secondary endpoint; however, the ability of HMGB1 to predict an increase in INR would be expected to translate into an ability to identify patients at risk of adverse outcomes such as acute liver failure and death. However, whether HMGB1 can predict adverse outcomes requires further validation in higher-risk populations. We speculate that the enhanced prognostic ability of HMGB1 in comparison with the other markers reflects its key role in the mechanism of injury. HMGB1 links hepatocyte death to the activation of an immune response by targeting Toll-like receptors and the receptor for advanced glycation end products.^33^, ^34^, ^35^ Blocking the effect of HMGB1 using anti-HMGB1 antibodies,^36^ inhibitory peptides,^37^ or by liver-specific HMGB1 gene ablation^11^ prevents the toxic effects of paracetamol in rodents. Anti-HMGB1 antibodies are being developed for human use and represent novel biological therapies for the treatment of acute liver injury.

We propose that miR-122 and HMGB1 offer a biomarker combination that is complementary to established markers. These new markers offer higher liver specificity (miR-122) and prognostic capability (HMGB1) that are fundamentally linked to the disease mechanism. Although we used continuous variables in our analysis, these findings now show the clinical validity of these biomarkers and provide a platform to assess the usefulness of cutoff values derived from these data in further prospective studies. Essential to the further development of these biomarkers is the availability of rapid and cost-effective clinical assays. Development and validation of clinical assays with a rapid turnaround time would allow their use for the stratification of patients presenting acutely with paracetamol overdose and for assessment of liver toxicity with other drugs. With further development, the measurement of one or more of these assays could be inexpensive and rapid. The protein analytes are in relatively high concentration in blood so would be amenable to measurement on existing hospital biochemistry platforms. With respect to miR-122, a 2017 report^38^ describes a rapid assay that can accurately diagnose liver toxicity and has a product profile suitable for use in the acute setting.

In the context of paracetamol overdose, the key finding of this study is that these markers identify patients who will require prolonged hospital treatment despite receiving acetylcysteine and irrespective of other clinical parameters. Therefore, they could facilitate clinical development of novel individualised acetylcysteine regimens and new therapeutic agents by enriching trials for patients who will develop injury. Currently acetylcysteine dosing is based solely on bodyweight despite multiple studies showing that the conventional dose (300 mg/kg) is too low in larger overdoses.^39^ miR-122 and HMGB1 could be used to refine patient selection for trials of higher acetylcysteine doses. New therapeutic agents—that target the unmet need of treating liver injury that develops despite acetylcysteine treatment—are in clinical development (eg, NCT03177395).^40^ These studies could be galvanised by making use of miR-122 and HMGB1, both for patient selection and as drug efficacy biomarkers. When effective therapeutic strategies are identified, miR-122, HMGB1, or a combination of both biomarkers could represent promising complementary diagnostics. These biomarkers might be particularly useful in staggered overdoses, where the use of acetylcysteine treatment nomograms is not recommended for selecting which patients require treatment, and when patients are being considered for hospital discharge at the end of acetylcysteine treatment. In support of this, we have published a proof-of-concept case report of a patient who developed clinically significant acute liver injury after overdose, but who had been discharged from hospital without acetylcysteine treatment because circulating ALT and paracetamol measurements did not indicate the need for treatment (ie, were false negative results). miR-122 and HMGB1 were significantly increased at first presentation to hospital in this patient and, therefore, their measurement in real time would have prevented this patient from developing liver injury by unequivocally indicating the need for acetylcysteine.^41^ Novel, short, acetylcysteine treatment regimens are in clinical development.^28^ Incorporation of miR-122, HMGB1, or K18 measurements into these treatment pathways (alongside ALT) has the potential to facilitate prompt patient discharge at the end of treatment and thus reduce hospital bed occupancy. For all these clinical indications it will be essential that future studies identify whether incorporation of biomarkers is cost-effective. Beyond paracetamol overdose, this prospective study supports the decision by the FDA and EMA to evaluate miR-122 and HMGB1 as tools for safety assessment in drug development.

In summary, these multicentre studies have shown the clinical validity of a panel of novel mechanism-based biomarkers in the context of patient stratification after paracetamol overdose. These biomarkers should be incorporated into future clinical trials to develop new therapeutic pathways for paracetamol overdose treatment. Furthermore, these liver injury markers should be considered for both retrospective and prospective analysis of the mechanistic basis of hepatotoxicity resulting from new compounds in development.

For the **Hmisc package** see http://CRAN.R-project.org/package=Hmisc

For **R software** see http://www.R-project.org/
