# Supplementary material for: Risk stratification after paracetamol overdose using mechanistic biomarkers: results from two prospective cohort studies
Source: Lancet Gastroenterol Hepatol. 2017 Nov 14;3(2):104–13. doi: 10.1016/S2468-1253(17)30266-2 (PMC5777094; doi:10.1016/S2468-1253(17)30266-2)
Supplement: Supplementary appendix [file mmc1.pdf]

THE LANCET
Gastroenterology & Hepatology

Supplementary appendix

This appendix formed part of the original submission and has been peer reviewed. We post it as supplied by the authors.

Supplement to: Dear JW, Clarke JI, Francis B, et al. Risk stratification after paracetamol overdose using mechanistic biomarkers: results from two prospective cohort studies. *Lancet Gastroenterol Hepatol* 2017; published online Nov 13. http://dx.doi.org/10.1016/S2468-1253(17)30266-2.

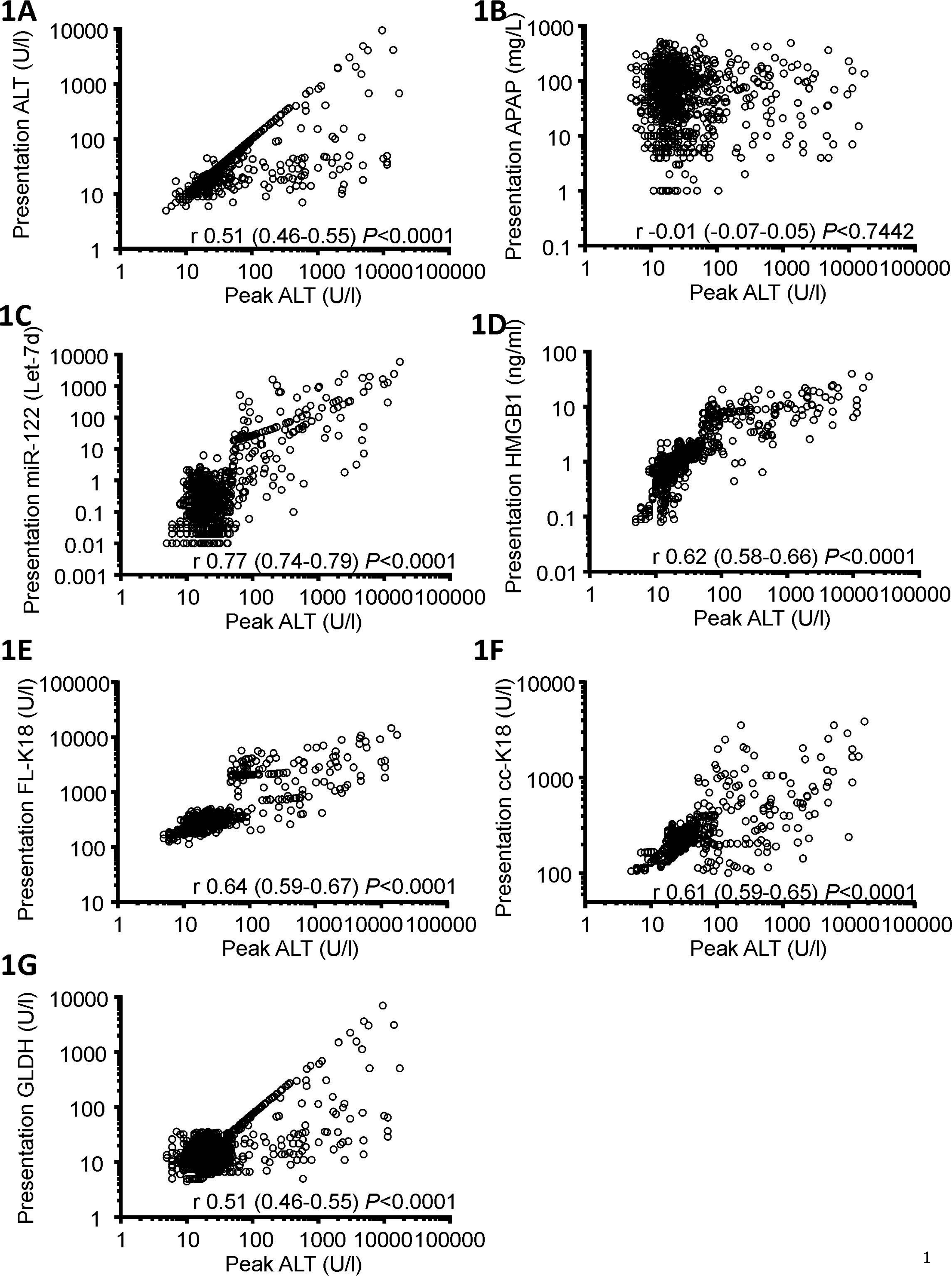
1A
Presentation ALT (U/l)
Peak ALT (U/l)
r 0.51 (0.46-0.55) P<0.0001
1B
Presentation APAP (mg/L)
Peak ALT (U/l)
r -0.01 (-0.07-0.05) P<0.7442
1C
Presentation miR-122 (Let-7d)
Peak ALT (U/l)
r 0.77 (0.74-0.79) P<0.0001
1D
Presentation HMGB1 (ng/ml)
Peak ALT (U/l)
r 0.62 (0.58-0.66) P<0.0001
1E
Presentation FL-K18 (U/l)
Peak ALT (U/l)
r 0.64 (0.59-0.67) P<0.0001
1F
Presentation cc-K18 (U/l)
Peak ALT (U/l)
r 0.61 (0.59-0.65) P<0.0001
1G
Presentation GLDH (U/l)
Peak ALT (U/l)
r 0.51 (0.46-0.55) P<0.0001

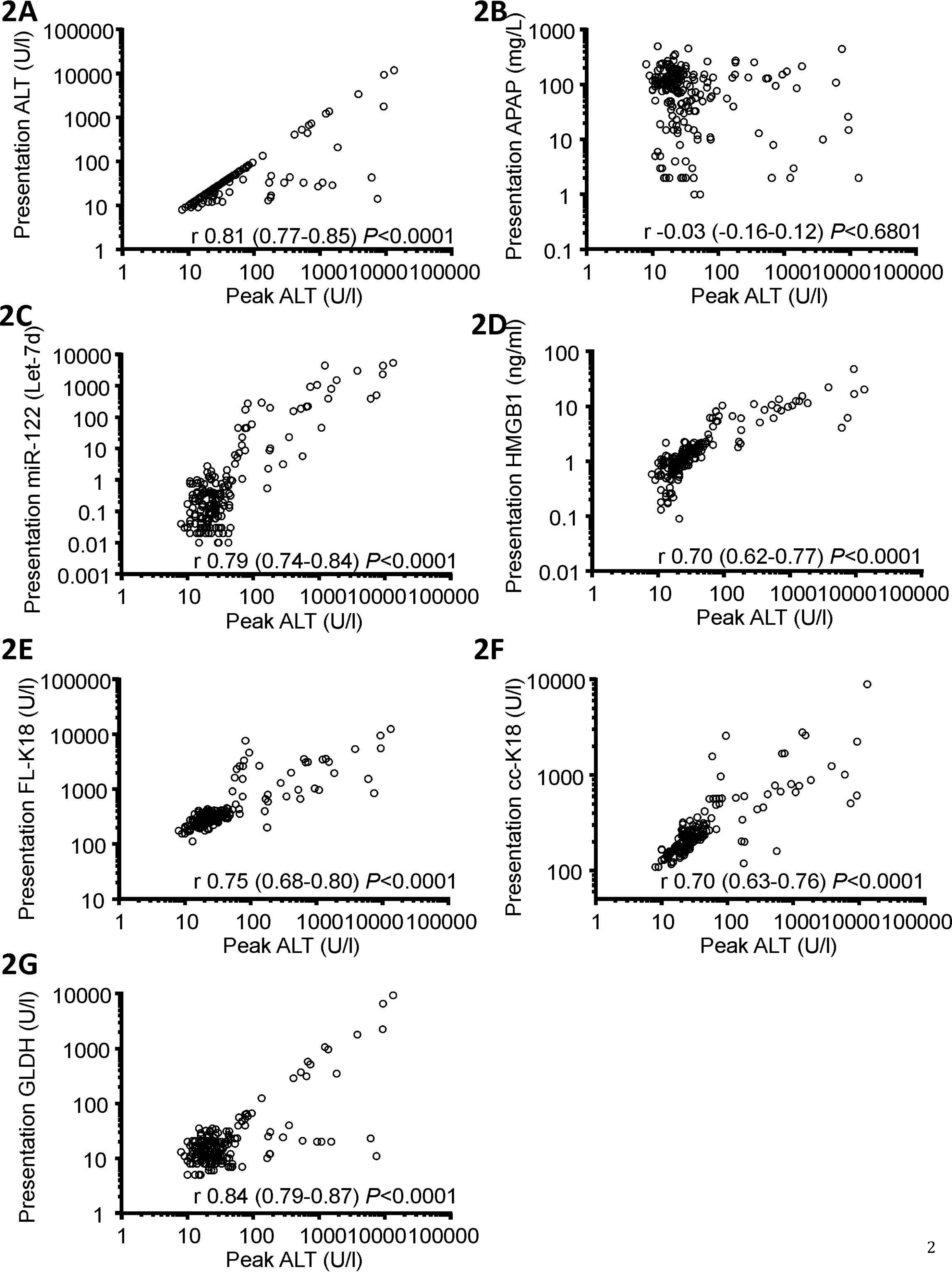
2A
Presentation ALT (U/l)
Peak ALT (U/l)
r 0.81 (0.77-0.85) P<0.0001
2B
Presentation APAP (mg/L)
Peak ALT (U/l)
r -0.03 (-0.16-0.12) P<0.6801
2C
Presentation miR-122 (Let-7d)
Peak ALT (U/l)
r 0.79 (0.74-0.84) P<0.0001
2D
Presentation HMGB1 (ng/ml)
Peak ALT (U/l)
r 0.70 (0.62-0.77) P<0.0001
2E
Presentation FL-K18 (U/l)
Peak ALT (U/l)
r 0.75 (0.68-0.80) P<0.0001
2F
Presentation cc-K18 (U/l)
Peak ALT (U/l)
r 0.70 (0.63-0.76) P<0.0001
2G
Presentation GLDH (U/l)
Peak ALT (U/l)
r 0.84 (0.79-0.87) P<0.0001

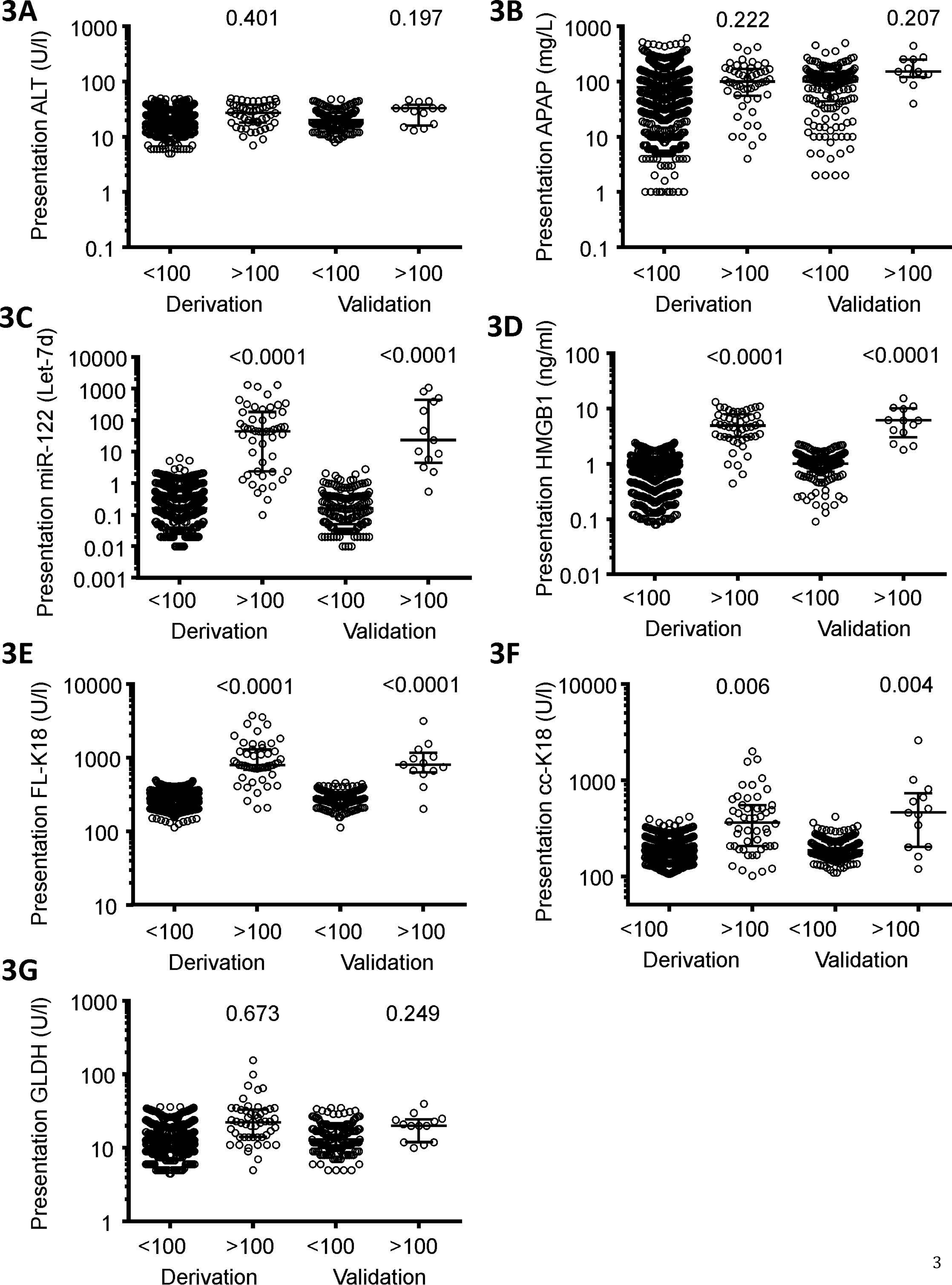
3A
Presentation ALT (U/l)
0.401
0.197
3B
Presentation APAP (mg/L)
0.222
0.207
3C
Presentation miR-122 (Let-7d)
<0.0001
<0.0001
3D
Presentation HMGB1 (ng/ml)
<0.0001
<0.0001
3E
Presentation FL-K18 (U/l)
<0.0001
<0.0001
3F
Presentation cc-K18 (U/l)
0.006
0.004
3G
Presentation GLDH (U/l)
0.673
0.249
<100
>100
<100
>100
Derivation
Validation

4A

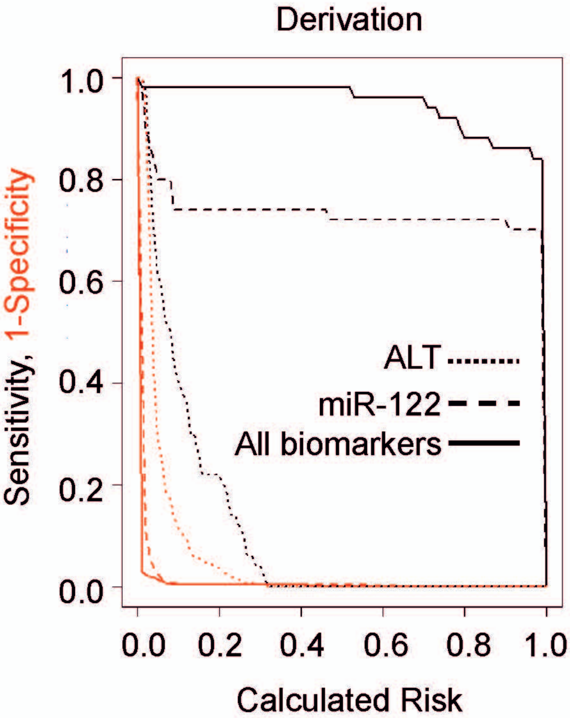
Derivation
Sensitivity, 1-Specificity
1.0
0.8
0.6
0.4
0.2
0.0
ALT
miR-122
All biomarkers
0.0 0.2 0.4 0.6 0.8 1.0
Calculated Risk

4B

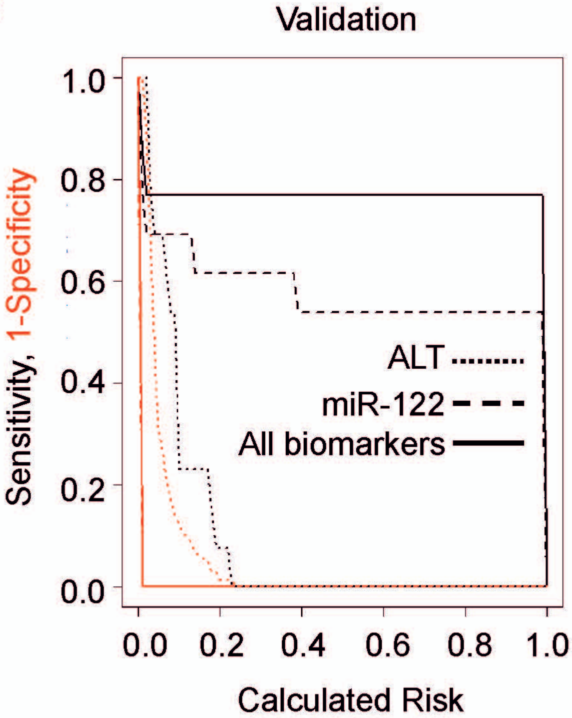
Validation
Sensitivity, 1-Specificity
1.0
0.8
0.6
0.4
0.2
0.0
ALT
miR-122
All biomarkers
0.0 0.2 0.4 0.6 0.8 1.0
Calculated Risk

4C

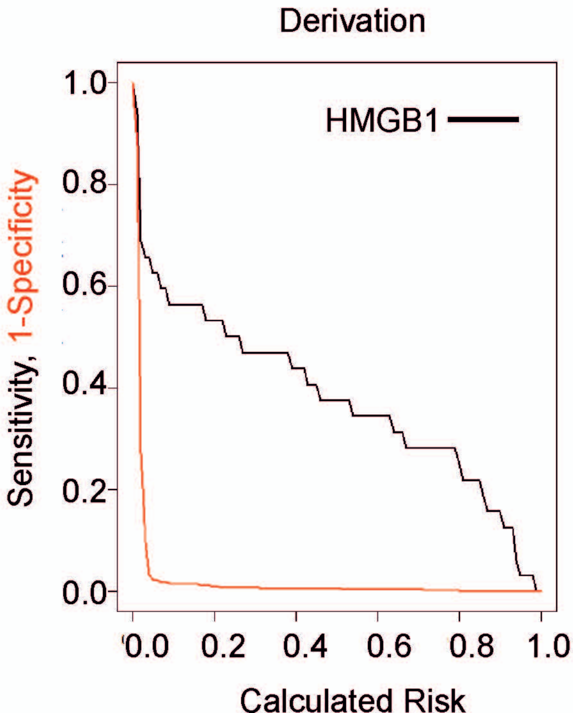
Derivation
Sensitivity, 1-Specificity
1.0
0.8
0.6
0.4
0.2
0.0
HMGB1
0.0 0.2 0.4 0.6 0.8 1.0
Calculated Risk

4D

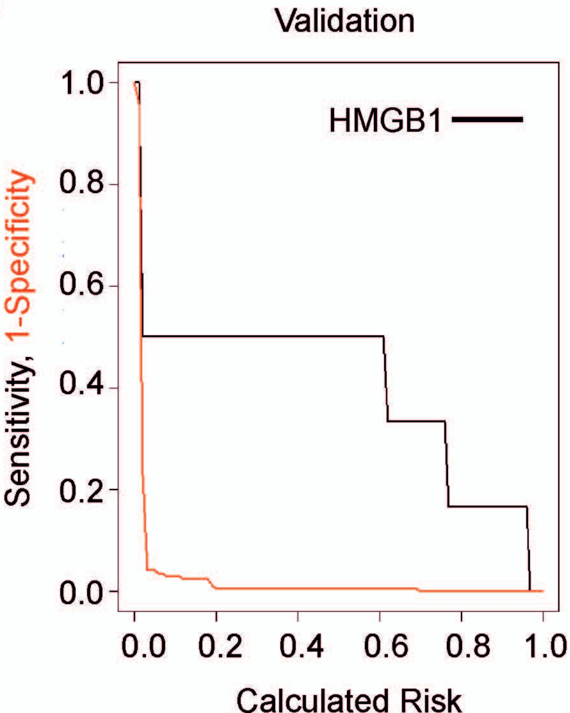
Validation
Sensitivity, 1-Specificity
1.0
0.8
0.6
0.4
0.2
0.0
HMGB1
0.0 0.2 0.4 0.6 0.8 1.0
Calculated Risk

Supplementary table 1:

When all patients recruited to both studies are included, novel biomarkers accurately predicted peak ALT >1000 U/l after APAP overdose. For the derivation cohort, N=28 developed ALT >1000 U/l from a total of 985. For the validation cohort N=11 developed ALT >1000 U/l from a total of 202.

**Biomarker**	**Derivation cohort (MAPP)**						**Validation cohort (BIOPAR)**					
	**ROC-AUC**	***P***	**Specificity**	**Sensitivity**	**PPV**	**NPV**	**ROC-AUC**	***P***	**Specificity**	**Sensitivity**	**PPV**	**NPV**
**ALT**	0.85 (0.78-0.93)	<0.0001	0.95	0.51 (0.34-0.68)	22.4	98.0	0.90 (0.81-0.98)	0.0003	0.95	0.57 (0.18-0.90)	43.7	97.8
**APAP concentration**	0.52 (0.41-0.64)	0.5760	0.95	0.06 (0.01-0.20)	3.5	95.7	0.65 (0.44-0.88)	0.0783	0.95	0.36 (0.11-0.70)	4.2	88.9
**miR-122**	0.97 (0.96-0.99)	<0.0001	0.95	0.84 (0.68-0.94)	28.1	99.3	0.99 (0.97-1.00)	<0.0001	0.95	0.90 (0.59-0.99)	52.6	99.5
**HMGB1**	0.97 (0.97-0.98)	<0.0001	0.95	0.78 (0.62-0.90)	27.1	98.6	0.98 (0.95-1.00)	<0.0001	0.95	0.81 (0.48-0.97)	47.4	98.9
**FL-K18**	0.96 (0.94-0.97)	<0.0001	0.95	0.65 (0.47-0.88)	25.3	98.6	0.97 (0.94-0.99)	<0.0001	0.95	0.63 (0.31-0.89)	41.2	97.8
**cc-K18**	0.92 (0.88-0.97)	<0.0001	0.95	0.75 (0.59-0.88)	26.9	99.0	0.97 (0.95-0.99)	<0.0001	0.95	0.90 (0.59-0.99)	52.6	99.5
**GLDH**	0.90 (0.85-0.95)	<0.0001	0.95	0.62 (0.45-0.78)	24.7	98.4	0.86 (0.72-0.98)	<0.0001	0.95	0.63 (0.31-0.89)	43.7	97.8

Supplementary table 2:

Biomarkers accurately predict peak ALT >1000 U/l in those patients that had a normal ALT and INR at hospital presentation. For the derivation cohort, N=18 developed ALT >1000 U/l from a total of 875. For the validation cohort N=4 developed ALT >1000 U/l from a total of 176.

Biomarker	Derivation cohort (MAPP)						Validation cohort (BIOPAR)					
	ROC-AUC	*P*	Specificity	Sensitivity	PPV	NPV	ROC-AUC	*P*	Specificity	Sensitivity	PPV	NPV
**ALT**	0.78 (0.65-0.91)	<0.0001	0.95	0.33 (0.13-0.59)	12.0	98.5	0.69 (0.41-0.98)	0.1770	0.95	0.25 (0.01-0.81)	9.1	98.2
**APAP concentration**	0.61 (0.48-0.75)	0.1008	0.95	0.06 (0.002-0.29)	4.3	98.1	0.65 (0.40-0.90)	0.3059	0.95	0.25 (0.01-0.81)	9.1	98.2
**miR-122**	0.99 (0.99-1.00)	<0.0001	0.95	0.94 (0.73-0.99)	27.8	99.8	0.99 (0.98-1.00)	0.0008	0.95	1.00 (0.40-1.00)	33.3	100.0
**HMGB1**	0.99 (0.99-1.00)	<0.0001	0.95	0.95 (0.91-1.00)	25.4	100.0	0.98 (0.97-1.00)	0.0009	0.95	1.00 (0.40-1.00)	28.6	100.0
**FL-K18**	0.99 (0.98-1.00)	<0.0001	0.95	0.94 (0.73-0.99)	29.3	99.8	0.99 (0.98-1.00)	0.0007	0.95	1.00 (0.40-1.00)	33.3	100.0
**cc-K18**	0.98 (0.96-1.00)	<0.0001	0.95	0.88 (0.65-0.99)	29.1	99.7	0.99 (0.98-1.00)	0.0007	0.95	1.00 (0.40-1.00)	33.3	100.0
**GLDH**	0.88 (0.79-0.96)	<0.0001	0.95	0.50 (0.26-0.74)	20.5	98.0	0.67 (0.46-0.88)	0.2375	0.95	0.00 (0.00-0.60)	0.0	97.6

Supplementary table 3:

Biomarkers accurately predict peak ALT >100 U/l in those that have a normal ALT and INR at hospital presentation – acute overdose patients only, separated by time. For the derivation cohort, N=490 presented <8h. For the validation cohort N=100 presented <8h.

Biomarker	Derivation cohort (MAPP)				Validation cohort (BIOPAR)			
	<8h		>8h		<8h		>8h	
	ROC-AUC	*P*	ROC-AUC	*P*	ROC-AUC	*P*	ROC-AUC	*P*
**ALT**	0.60 (0.44-0.78)	0.1555	0.88 (0.81-0.95)	<0.0001	0.61 (0.62-0.81)	0.0522	0.82 (0.79-0.91)	<0.0001
**APAP concentration**	0.71 (0.62-0.83)	0.0025	0.67 (0.53-0.81)	0.0537	0.62 (0.53-0.76)	0.2604	0.59 (0.42-0.67)	0.0021
**miR-122**	0.92 (0.84-1.00)	<0.0001	0.98 (0.96-1.00)	<0.0001	0.90 (0.81-1.00)	<0.0001	0.96 (0.94-1.00)	<0.0001
**HMGB1**	0.85 (0.71-1.00)	<0.0001	0.98 (0.95-1.00)	<0.0001	0.88 (0.79-1.00)	<0.0001	0.95 (0.92-1.00)	<0.0001
**FL-K18**	0.92 (0.82-1.00)	<0.0001	0.95 (0.86-1.00)	<0.0001	0.91 (0.79-1.00)	<0.0001	0.94 (0.90-0.97)	<0.0001
**cc-K18**	0.74 (0.55-0.93)	<0.0001	0.90 (0.81-0.98)	<0.0001	0.70 (0.59-0.89)	<0.0001	0.88 (0.82-0.92)	<0.0001
**GLDH**	0.66 (0.45-0.83)	0.0382	0.83 (0.74-0.92)	<0.0001	0.63 (0.41-0.78)	0.0054	0.80 (0.72-0.89)	<0.0001

Supplementary table 4:

cfNRI and INI analysis confirmed the added value of novel biomarkers in both cohorts to accurately predict later peak ALT >100 U/l in those that have a normal ALT and INR at hospital presentation

Cohort	Model	cfNRI Event	cfNRI Non-Event	cfNRI Total	*P*	IDI Sensitivity	IDI Specificity	IDI Total	*P*
**Derivation (MAPP)**	miR-122	0.68 (0.48-0.88)	0.93 (0.91-0.96)	1.61 (1.41-1.81)	<0.001	0.63	0.04	0.67 (0.55-0.78)	<0.001
	+FL-K18	0.72 (0.53-0.91)	0.93 (0.91-0.96)	1.65 (1.46-1.85)	<0.001	0.63	0.04	0.67 (0.55-0.78)	<0.001
	+cc-K18	0.92 (0.81-1.03)	0.97 (0.96-0.99)	1.89 (1.78-2.00)	<0.001	0.81	0.05	0.86 (0.78-0.93)	<0.001
	+HMGB1	0.96 (0.88-1.04)	0.99 (0.98-1.00)	1.95 (1.87-2.03)	<0.001	0.84	0.05	0.89 (0.84-0.94)	<0.001
**Validation (BIOPAR)**	miR-122	0.39 (-0.12-0.89)	1.00 (1.00-1.00)	1.39 (0.88-1.89)	<0.001	0.49	0.05	0.54 (0.28-0.80)	<0.001
	+FL-K18	0.39 (-0.12-0.89)	1.00 (1.00-1.00)	1.39 (0.88-1.89)	<0.001	0.47	0.05	0.53 (0.26-0.79)	<0.001
	+cc-K18	0.69 (0.30-1.08)	1.00 (1.00-1.00)	1.69 (1.30-2.08)	<0.001	0.70	0.05	0.75 (0.54-0.96)	<0.001
	+HMGB1	0.54 (0.08-1.00)	1.00 (1.00-1.00)	1.54 (1.08-2.00)	<0.001	0.68	0.05	0.73 (0.51-0.96)	<0.001

Supplementary table 5:

Number of correctly diagnosed patients using new cfNRI-based model. All patients had a normal ALT and INR at hospital presentation but either did or did not develop ALI as defined by peak ALT >100 during the hospital stay.

Model	Derivation cohort (MAPP)		Validation cohort (BIOPAR)	
	Later ALI Correctly Identified	Later Non-ALI Correctly Identified	Later ALI Correctly Identified	Later Non-ALI Correctly Identified
miR-122	45/50	735/825	9/13	163/163
+necrosis.K18	47/50	824/825	10/13	163/163
+apoptosis.K18	48/50	812/825	11/13	163/163
+HMGB1	49/50	824/825	10/13	163/163
